# Evaluation of Local Tissue Reaction After the Application of a 3D Printed Novel Holdfast Device for Left Atrial Appendage Exclusion

**DOI:** 10.1007/s10439-019-02320-2

**Published:** 2019-07-15

**Authors:** Maciej Brzeziński, Aleksandra Sejda, Rafał Pęksa, Maciej Pawlak, Kamil Bury, Zbigniew Adamiak, Maciej Kowalik, Dariusz Jagielak, Krzysztof Bartus, Mateusz K. Hołda, Radoslaw Litwinowicz, Jan Rogowski

**Affiliations:** 1 Department of Cardiac and Vascular Surgery, Medical University of Gdansk, Gdańsk, Poland; 2 Department of Pathomorphology, Medical University of Gdansk, Gdańsk, Poland; 3 Department of Surgery and Roentgenology, Faculty of Veterinary Medicine, University of Warmia and Mazury, Olsztyn, Poland; 4 Department of Anesthesiology and Intensive Care, Medical University of Gdansk, Gdańsk, Poland; 5 Department of Cardiovascular Surgery and Transplantology, Jagiellonian University Medical College, Kraków, Poland; 6 HEART - Heart Embryology and Anatomy Research Team, Department of Anatomy, Jagiellonian University Medical College, Kopernika 12, 31–034 Kraków, Poland

**Keywords:** Stroke prevention, 3D printing, Cardiovascular surgery, Atrial fibrillation, Left atrium

## Abstract

The left atrial appendage (LAA) is a small, finger-like extension of the left atrium and its exclusion is used as a treatment strategy to prevent ischemic stroke. Existing holdfast devices may damage the tissue, are unisized and not adjustable. A novel holdfast device for LAA exclusion devoid of these shortcomings was designed and 3D-printed using the Selective Laser Sintering (SLS) technology with polyamide powder and tested it on animal model. We selected the SLS 3D printing technology due to its wid14e availability and low production costs which could provide on-site 3D printing for specific patient. The purpose of this study was to evaluate the biocompatibility of the reported holdfast device and compare the histological results obtained for local tissue reactions to those obtained for an established grafting material. Thirty swine subdivided into two groups were examined. The LAA exclusion device was implanted and was either coated with a polyester vascular implant or not coated at all and the histological response to the device’s presence was evaluated which is a standard approach to test the device biocompatibility. In all cases, complete occlusion was seen without any pathological findings during the incubation time. In both groups, the surface of the atrium under a holdfast device was smooth and shiny and had no clots. The foreign body reaction of the LAA holdfast device made of polyamide powder was insignificantly lower compared to the polyester graft. Thus, it fulfils the parameters of biocompatibility at the highest degree, and makes it suitable material for the manufacturing of LAA holdfast devices.

## Introduction

**Figure 1:**
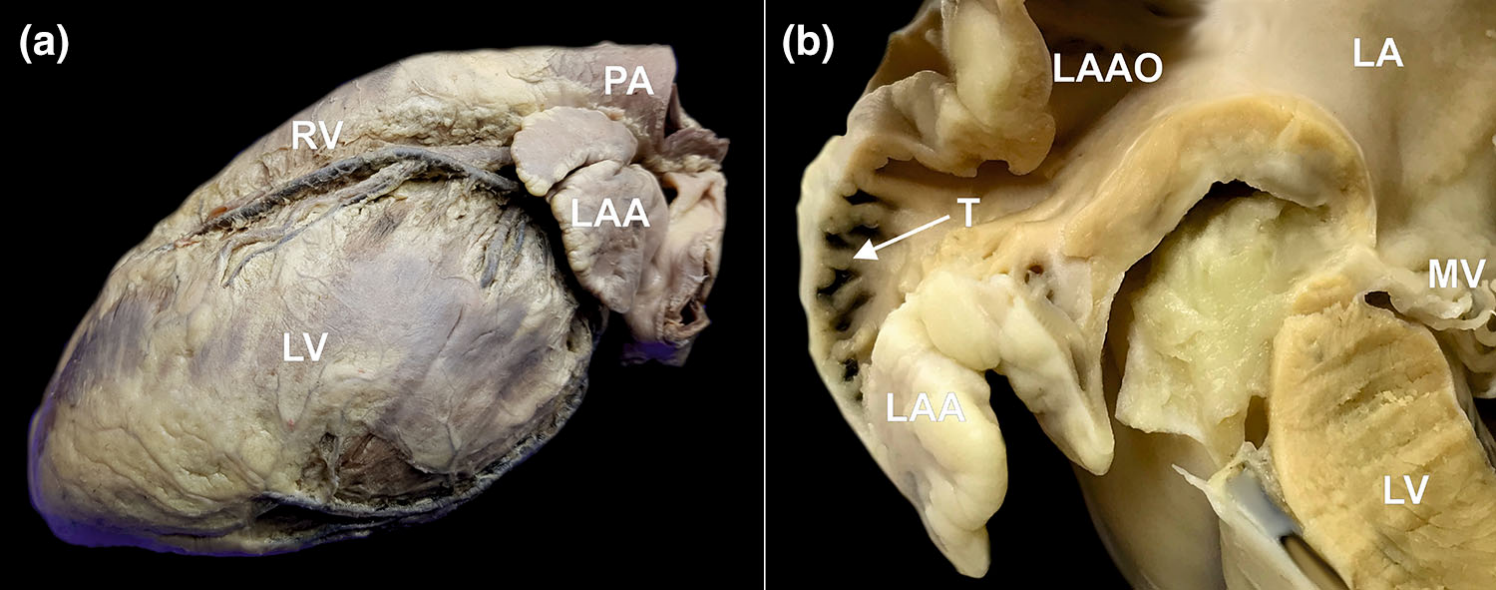
Photographs of human cadaveric heart specimens showing left atrial appendage (LAA). (**a**) The left lateral view on the heart showing the location of the LAA. (**b**) Cross section through the left atrium (LA) and the LAA. Narrow left atrial appendage ostium (LAAO) and rich trabeculations (T) within the LAA are visible, which are responsible for blood stasis and clot formation. *MV* mitral valve, *LV* left ventricle, *PA* pulmonary artery, *RV* right ventricle.

The left atrial appendage (LAA) is a small, finger-like extension of the left atrium protruding from its lateral or inferolateral wall with a narrow junction to the venous component of the left atrium (Fig. 1a). Embryologically, it is a remnant of the original left atrium, and in contrast to the smooth walled venous component of the left atrium, it is richly trabeculated (Fig. 1b). The LAA is considered largely nonfunctional. However, its complex morphology, trabeculations, narrow ostium and scant blood flow make the LAA a prime cardiac site for thrombus formation. Patients with atrial fibrillation are particularly at risk, and complications may lead to ischemic stroke.^2^,^31^ Atrial fibrillation is the most frequent type of cardiac arrhythmia, although it maintains a low rate of successful treatment.^9^ Patients with atrial fibrillation have a five-fold greater risk for developing ischemic stroke due to formation of blood clots in their LAAs.^30^ This may occur incidentally or rapidly (within 6 h of onset of symptoms) in some cases.^6^ The most common method for preventing ischemic stroke is antithrombotic treatment. However, pharmacological treatments pose a risk of major bleeding, including intracranial hemorrhage.^4^ Additionally, antithrombotic therapy is not recommended for some patients and may even be prohibited as a primary treatment modality in some cases.^4^,^21^,^24^ A new approach to solve the abovementioned issues would consist of obliterating the LAA, which remains the most frequent source of clot formation within the heart.^7^,^19^,^20^,^22^

To date, there are several devices on the market that can successfully occlude the LAA.^7^,^13^,^18^–^20^,^22^,^23^ There are two categories of LAA closure devices which are designed to close the base of the LAA and prevent blood flow to circulate through it (thus excluding the LAA from the circulatory system). They are classified by their method of implantation. There are holdfasts which are introduced by performing a thoracotomy to close the LAA from the outside, and excluders, which are placed by using endovascular techniques to close the LAA from the inside. The advantage of the holdfasts is their great efficiency, whereas the latter have a less invasive procedure (the procedure is performed under local anesthesia with transcatheter and no surgical opening of the chest).^5^

There are several existing holdfast devices on the market, however each of them has some disadvantages. Some of them (particularly staplers) are designed to pierce the base of the LAA during implantation. However, this initial step damages the tissue and interrupts the continuity of the left atrial wall and leads to blood leakage.^14^ Moreover, currently used devices do not allow post-insertion adjustments after release from the applicator. Finally, they are produced in a single size without possibility for modifications for the specific anatomy of a given patient.^8^ Considering the advantages and disadvantages of the available LAA closure devices, we designed a novel holdfast device for LAA obliteration devoid of these shortcomings.^10^

The aim of this study was to evaluate the biocompatibility of the proposed device and examine the morphological and histological processes occurring in the adjacent tissues after the implementation of the device. We paid particular emphasis on the type and extent of inflammatory reactions occurring during proper tissue cicatrization. We also assessed the possible gross pathologies and side effects that could result from the implantation of our device.

## Materials and Methods

### Device Design

**Figure 2:**
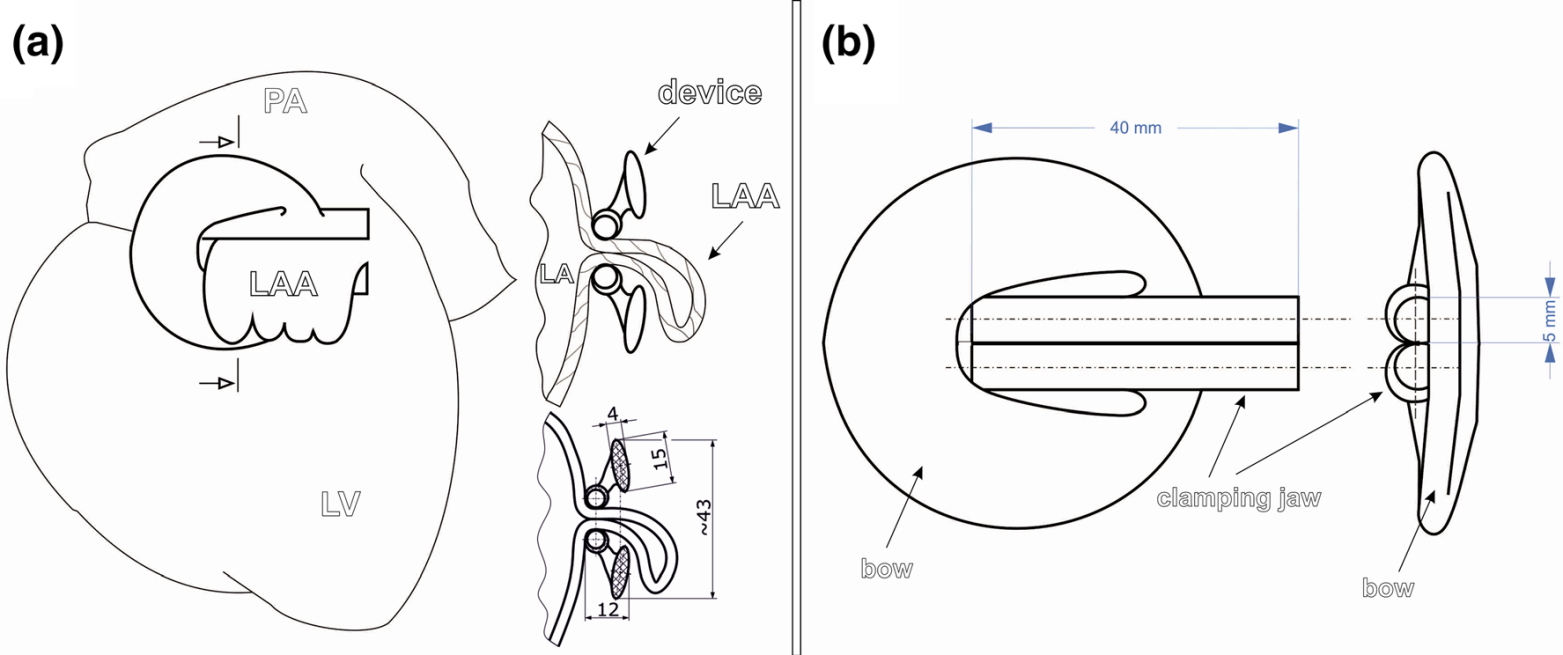
Schematic image of the left atrial appendage (LAA) holdfast device. (**a**) Left lateral view on the heart with implanted LAA holdfast device and a cross section through the LAA excluded from the left atrium (LA) by the holdfast device is showed. Dimensions of the LAA holdfast device are reported in mm. (**b**) Technical drawing of the designed LAA holdfast device. *LV* left ventricle, *PA* pulmonary artery.

To occlude the LAA from the outside and thus prevent the connection between the left atrium and the appendage without damaging the surrounding tissue, a special holdfast device was designed in cooperation with the Technical University of Gdańsk, Poland. The construction of the holdfast was the subject of a Patent Cooperation Treaty, which is registered under No. PCT/PL2014/000031. The device consisted of two tubes connected with an elastic bow (Fig. 2). The concept of operation for the device was based on the idea that the tubes would press the LAA base, which would lead to its closure and occlude the connection between the LAA and left atrium (Fig. 2). Moreover, the intention was that the device could be freely positioned around the LAA and could be easily adjusted after implantation.

The whole unit was made monolithically using a 3D printing method with Selective Laser Sintering (SLS) technology with polyamide powder PA 2200 (PA 12), which is a biostable polymer that is used in biomedical engineering. The biocompatibility of the material was certified according to ISO 10993-1.^16^ The polyamide powder is widely used in clinical medicine and until now has shown no carcinogenicity.^26^ Polyamides are long-chain polymers with amide bonds that are the result of the reaction of acids with amines. Due to a good balance between properties (high strength and rigidity, good chemical resistance, high long-term stability, good selectivity resolution and true to detail) and its low price, they are one of the most common polymers applied.^17^ Products made of PA 12 have near-perfect chemical and thermal resistance, low moisture absorption and high resistance to deformation. The SLS 3D printing technology was selected due to its widespread availability and low production costs which could allow future on-site 3D printing for specific patient.

The dimensions of the clip were calculated, designed and described by computer modelling (finite element method) keeping in mind the stretch and thickness of a human LAA, which is approximately 6 mm, and were thus designed to have a clamping force of 36 N. This force value was found to be advantageous for LAA occlusion in a large canine model.^13^ From engineering point of view, the target to achieve was a low stiffness spring with a highly degressive characteristic of the device. This was achieved by: choice of material (PA 2200 polyamide with Young’s modulus of 1500 MPa, Poisson’s ratio of 0.38, tensile yield strength of 45 MPa and elongation at break of 24%) and shifting the bow crossection axis away from the device axis (which results in bending at an arm that grows as the spring is deflected).

**Figure 3:**
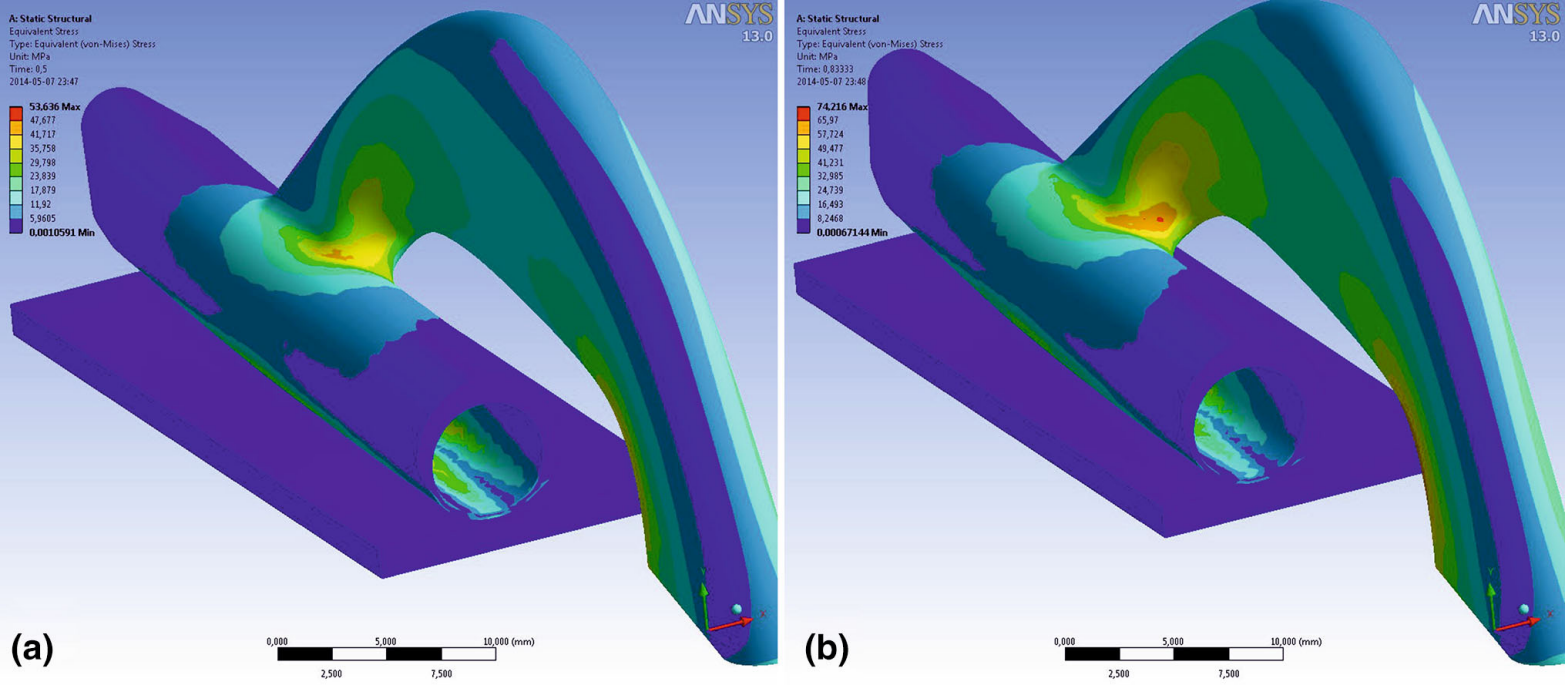
Stress distribution in the holdfast device at (**a**) substantial deflection caused by a left atrial appendage of 6 mm in thickness and (**b**) substantial deflection of 10 mm (maximum value, limited by application tool geometry).

Choice of geometric parameter of the spring was achieved by multivariant topology corrections in Finite Element Method (FEM) calculations. FEM model in ANSYS Workbench software was designed as symmetric half of the spring, one wall of left atrial appendage of width 1.5 mm (Young’s modulus 9 MPa, Poisson’s ratio 0.4) and their interaction* via* contact finite elements. Model load on holdfast device (supported on plane of symmetry) was deformation in *Y* direction of the lower wall of the model up to 6 mm (corresponding to whole spring deformation of 12 mm). As a result of multiple trials, a sufficiently uniform stress distribution was achieved. This stress is of high value, which might render stress relaxation after a few months of device implantation, when irreversible changes in tissue occur and the pressure would not be needed anymore. At maximum deflection of the spring (limited by application tool geometry) of 10 mm the device undergoes local plastic deformation, only in flexible joint—connection the bow and tubes. The maximum extension of the tubes was set to 10 mm—this value was enough to help with the implementation process but would still not pose a hazard to the structure of the device (Fig. 3).

### *In Vivo* Animal Testing

**Figure 4:**
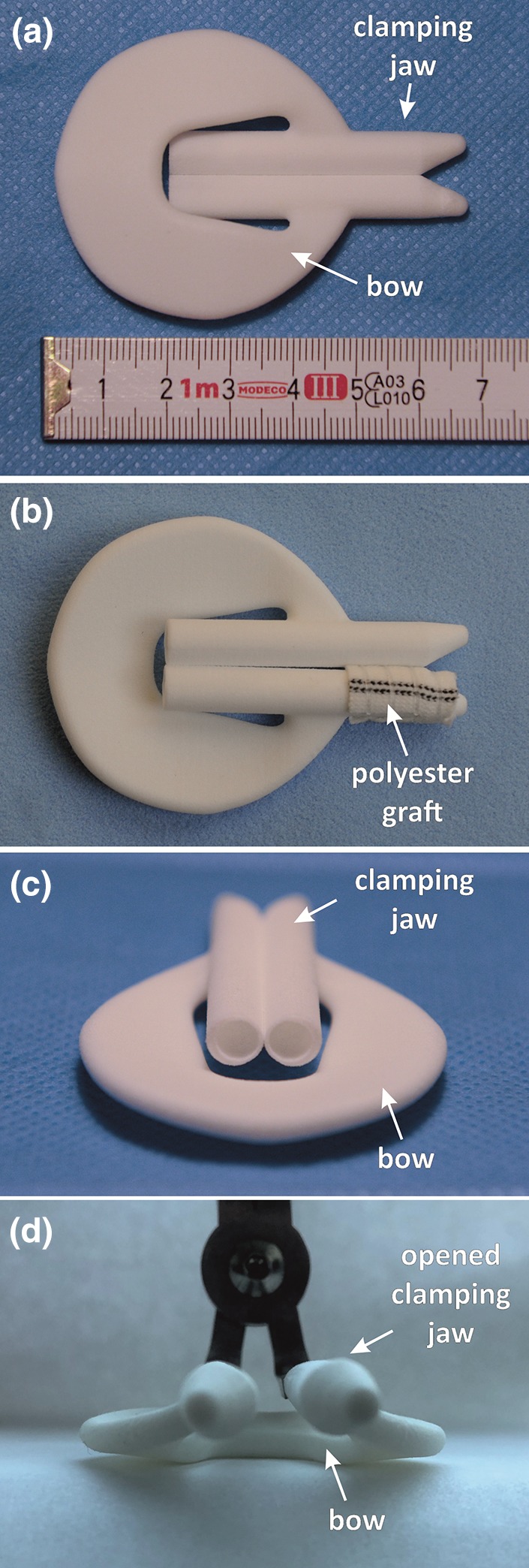
Holdfast device (**a**) without additional materials implanted in group I and (**b**) coated with a vascular graft (polyester Vascutek Gelsoft Prosthesis, Terumo, Scotland, UK) implanted in group II. (**c**) The side view on the holdfast device. (**d**) Holdfast device positioned on the applicator with opened clamping jaw.
- Group II, had one tube of the holdfast device coated with a vascular implant, Vascutek Gelsoft Prosthesis, Terumo (Scotland, UK), which was made of polyester (Fig. 4b).

After obtaining the end-product prototype, which met all the necessary criteria of an LAA holdfast (clamping force of 36 N, a size which would allow to close the base of the LAA, the possibility of increased spread of the clamping jaw up to 10 mm without damaging the clamp), we conducted an *in vivo* investigation of its effectiveness and safety. This part of study was conducted in collaboration with the Faculty of Veterinary Medicine at Warmia and Mazury University, and the experiments were performed in a swine model. Two study groups were formed to assess the reaction between the material of the holdfast and a material with a well-known and documented local reaction in the human body (polyester vascular prosthesis Vascutek Gelsoft Prosthesis, Terumo, Scotland, UK):

- Group I, had the standard, uncoated holdfast device without additional materials applied (Fig. 4a),

This inquiry was consistent with the European Standard pertaining to “Biological evaluation of medical devices—Part 6. Test for local effects after implantation” (ISO 10993-6:2007).^15^ All procedures were executed with strict accordance with the recommendations of the Guide for the Care and Use of Laboratory Animals of the National Institutes of Health. The protocol was approved by the Committee on the Ethics of Animal Experiments of the Medical University of Gdańsk, Poland (Permit Number: 42/2013) and the Faculty of Veterinary Medicine at Warmia and Mazury University, Poland (Permit Number: 21/S/2013). All efforts were made to minimize the suffering of the animals. The protocols for anesthesia and animal sacrifice were also approved by both Universities.

**Figure 5:**
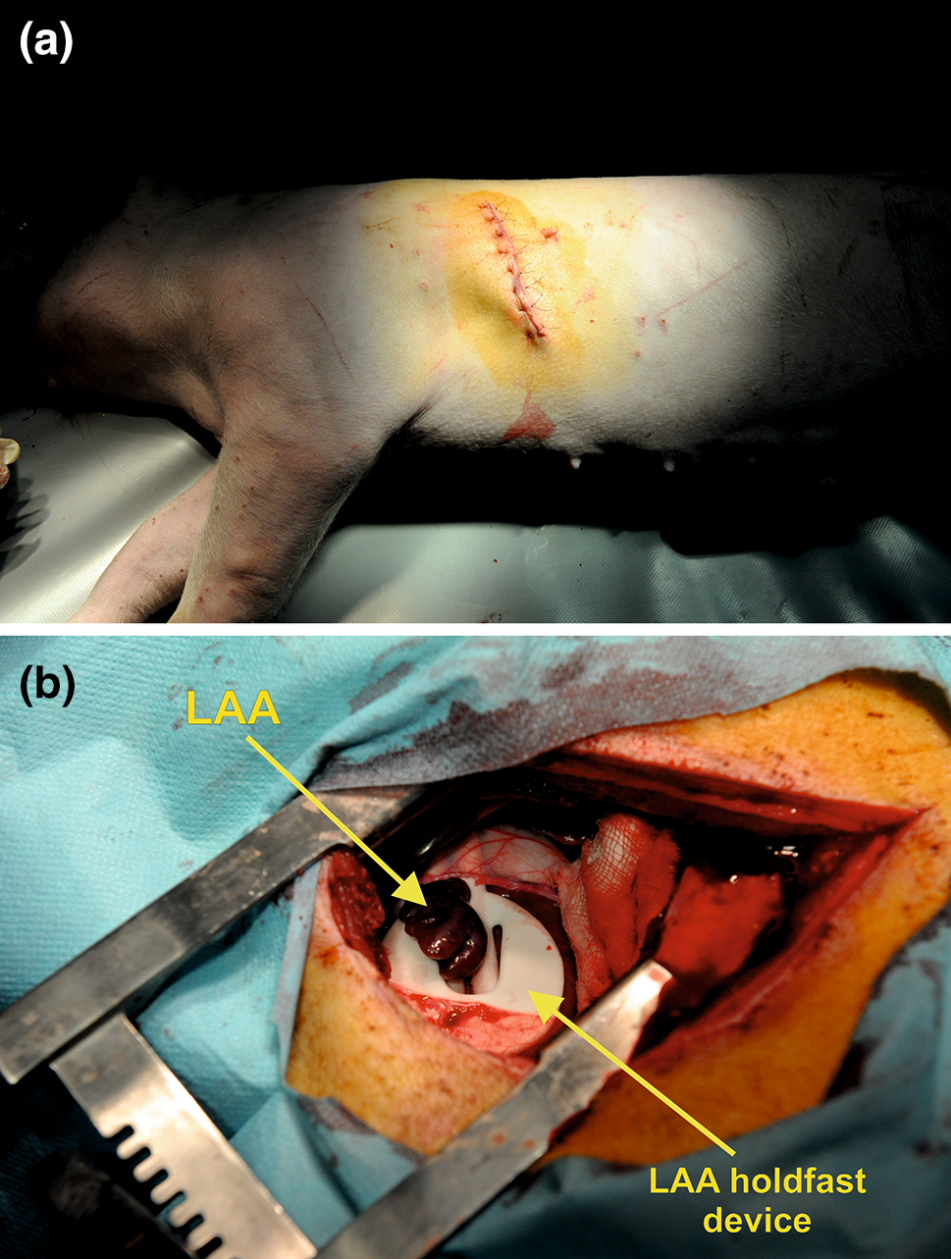
Periprocedural photographs showing (**a**) access site* via* left thoracotomy (the chest was opened at the fourth intercostal space) and (**b**) implanted left atrial appendage (LAA) holdfast device.

For the experiment, 30 swine (*Sus scrofa f. domestica, Great white Polish pig*, 60% females, 17 weeks old) were used. The animals came from one breed and had an average body weight of 55.5 kg (range 55.0–60.0 kg). After 14 days of quarantine, the animals were randomly divided into two study groups (15 swine in each group) and subjected to device implantation. Routine general anesthesia was used. Access to the mediastinum was obtained* via* left thoracotomy. The chest was opened at the fourth intercostal space (Fig. 5a). All holdfast devices were sanitized in 0.5% Aniosyme DD1 (Laboratories Anios, France) for a period of 5 min and at a temperature of 20 °C. After drying the devices, they were sanitized in 100% ethylene oxide at a temperature of 55 °C for 60 min. The holdfast devices were implanted after a minimum of 12 h of aeration. The application was made identically in both groups, according to the previously established procedure (Fig. 5b).^10^

On the 14th day after the surgical procedure, the animals were subjected to euthanasia, except for four animals from group II, which were subjected to a prolonged incubation. To fulfill the EN ISO 10993-6:2009 IDT recommendations for the prolonged observation of the tissue reaction in the comparison model, the incubation in these four swine lasted for 90 days.^15^ To perform the euthanasia, routine general anesthesia was performed and pentobarbital sodium (Euthasol vet, Le Vet B.V., Netherlands) was delivered intravenously in a dose of 140 mg/kg of body weight. Immediately after the animals’ euthanasia, hearts were dissected in a routine manner, washed out of blood and fixed by immersion in 4% paraformaldehyde buffered solution for a 48 h period.

### Postmortem Evaluation

The hearts were subjected to a macroscopic evaluation and observed to see whether there was formation of adhesions between the occluded LAA walls. They were also examined to evaluate the tissue reaction to polyamide powder at the Pathomorphology Institute, Medical University of Gdańsk, Poland. In all 30 swine hearts, the efficacy of the LAA occlusion was checked (this was determined by assessing the tightness in the clamp line and the presence of the tissue concrescence). Next, fragments of the left atrial wall with the appendage and the novel holdfast device were sliced into 23.0 × 30.0 × 5.0 mm^3^ blocks. Each block consisted of a LAA with an intersection through the holdfast device. Next, the samples were subject of a standard histological examination. In short, specimens were subsequently dehydrated by administering appropriate concentrations of ethanol alcohol and xylene and were then embedded in paraffin. All paraffin blocks were cut into 4.0 *μ*m thick serial sections. The samples were dyed with hematoxylin and eosin according to routine procedure.

To assess the tissue reaction to the implanted LAA device, the following were examined for each swine in both study groups:

- hyperemia: 0—lack, 1—small degree, 2—medium degree, 3—significant degree,
- intensity of chronic inflammatory infiltrate (measured by counting the number of lymphoid mononuclear cells): 0—lack, 1—small degree, 2—medium degree, 3—significant degree of chronic inflammatory infiltration,
- presence of purulent infiltration: 0—lack, 1—present,
- presence of foreign-body giant cells: 0—lack, 1—present,
- and the degree of cicatrization: 0—lack, 1—small degree, 2—medium degree, 3—significant degree.

### Statistical Analysis

Categorical variables were expressed as counts and percentages. The Shapiro–Wilk test was performed to determine normal distribution. When comparing continuous variables between groups that had a normal distribution, an independent *t-*test was used. Continuous variables that did not exhibit a normal distribution were compared with a Mann-Whitney *U* test. All dichotomous variables were compared using *x*^2^ analysis or Kolmogorov–Smirnov test. The sample size was estimated assuming that to show difference between 10 and 50% in single parameter change requires at least 20 animals with 80% statistical power and a significance level of 0.05 (two-sided test). Statistical analysis was performed with STATISTICA 12.0 (StatSoft, Tulsa, OK, USA). A two-sided p-value lower than 0.05 was considered statistically significant.

## Results

**Figure 6:**
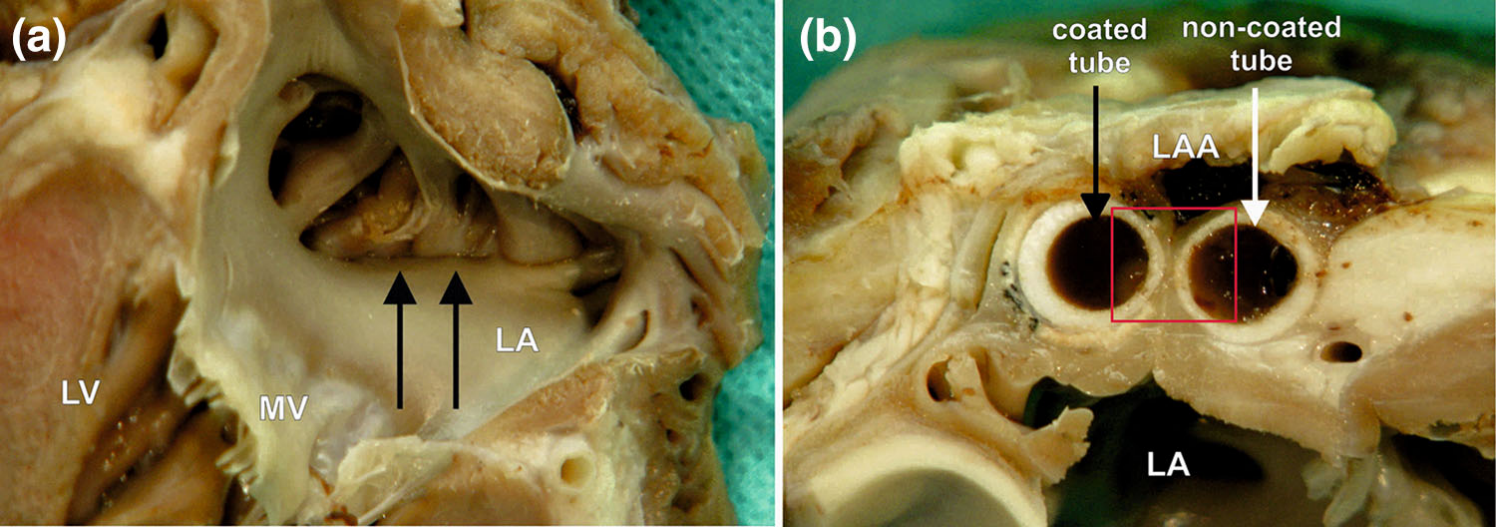
A macroscopic view of the left atrial appendage (LAA) closed with the holdfast device in group II (one tube coated with vascular implant). (**a**) The inner surface of the left atrium (LA) and (**b**) the intersection through the area of a holdfast device is shown. (**a**) Black arrows indicate the line of atrial wall adhesion placed between tubes of holdfast device. (**b**) The white arrow indicates the tube of holdfast device without and black arrow with a vascular implant. Red square indicates area showed in Fig. 8. *MV* mitral valve, *LV* left ventricle.

There were no perioperative deaths and a full recovery with complete physiological activity was observed in all animals. In all subjects, a complete LAA occlusion was obtained. In both tested groups (including swine with prolonged incubation time), the surface of the atrium under a holdfast device was smooth and shiny and lacked clots and the walls of the LAA located between the holdfast device closely adhered to each other. The line of the appendage wall’s adhesion was not fully formed, and the left appendage did not undergo total atrophy. On the surface between and around the holdfasts, fibrous connective tissue was visible (Fig. 6). Based on the macroscopic evaluation, no crucial differences in tissue response to different device tubes were observed.

**Figure 7:**
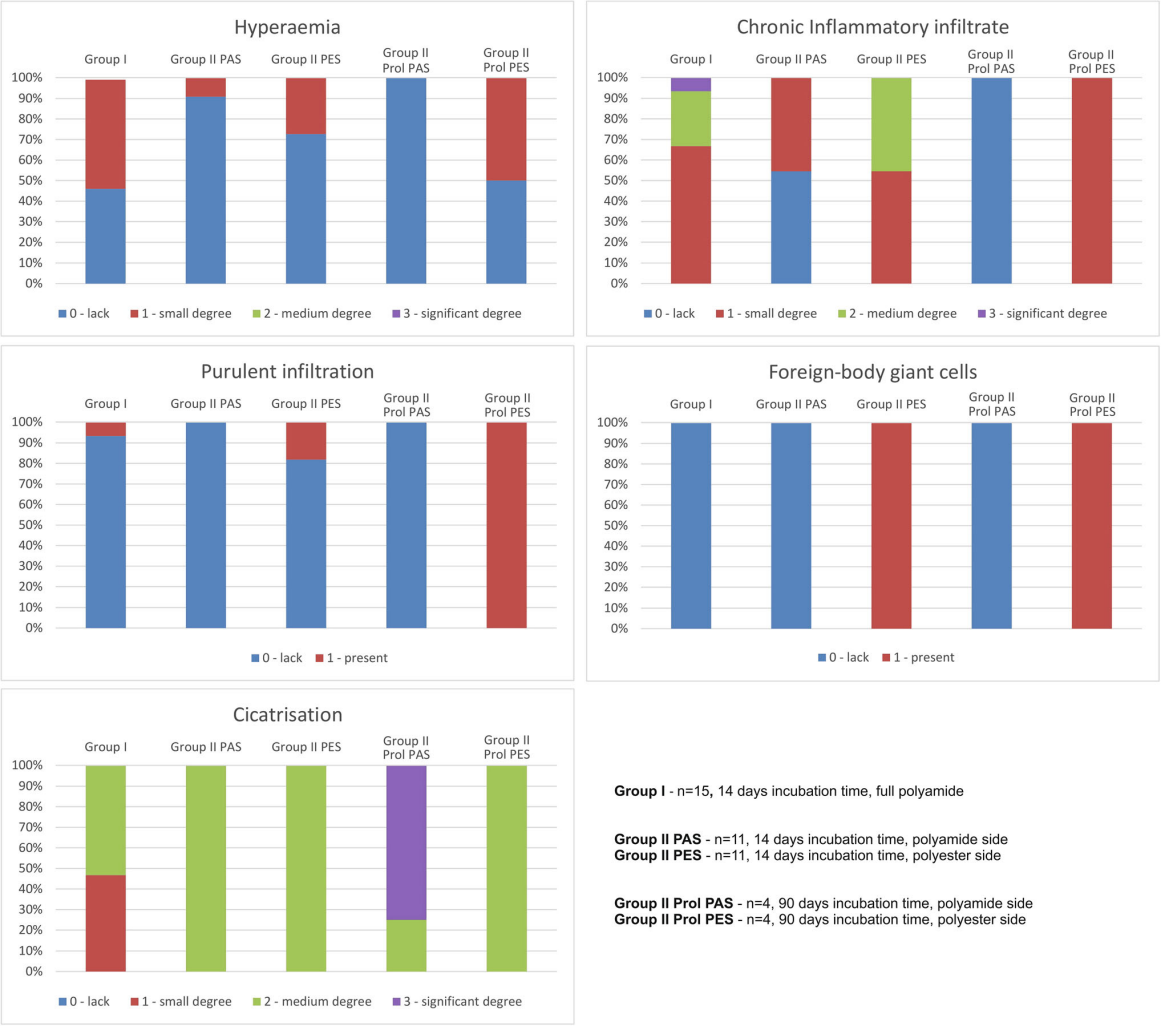
Column charts showing comparison of histological data between study groups.

Table 1 and Fig. 7 show the between-groups comparisons of all histological findings. No signs of holdfast material degeneration were observed in any study group.

**Table 1:** The comparison of histological data between study groups.

Examined feature	Group I n = 15 (% of incidence, full polyamide)	Group II		p-value Group I vs. Group II 14 days polyamide side	p-value Group I vs. Group II 90 days polyamide side	p-value Group I vs. Group II 14 days polyester side	p-value Group I vs. Group II 90 days polyester side	p-value Group II 14 days polyamide side vs. Group II 14 days polyester side	p-value Group II 90 days polyamide side vs. Group II 90 days polyester side
		14 days incubation time n = 11 (% of incidence on the polyamide side/% of incidence on the polyester side)	90 days incubation time n = 4 (% of incidence on the polyamide side/% of incidence on the polyester side)						
Hyperaemia									
0—lack	46.2	90.9/72.7	100.0/50.0	0.1771	0.3539	0.6564	0.5804	0.0542	0.4142
1—small degree	53.8	9.1/27.3	0/50.0						
2—medium degree	0	0/0	0/0						
3—significant degree	0	0/0	0/0						
Chronic inflammatory infiltrate^a^									
0—lack	0	54.5/0	100.0/0	**0.0003**	0.4649	0.4046	**0.0039**	**0.0063**	**0.0339**
1—small degree	66.7	45.5/54.5	0/100.0						
2—medium degree	26.7	0/45.5	0/0						
3—significant degree	6.7	0/0	0/0						
Purulent infiltration									
0—lack	93.3	100.0/81.8	100.0/100.0	0.4657	0.7743	0.4657	0.4583	0.8738	0.7237
1—present	6.7	0/18.2	0/0						
Foreign-body giant cells									
0—lack	100.0	100.0/0	100.0/0	–	–	**< 0.0001**	**< 0.0001**	**< 0.0001**	**< 0.0001**
1—present	0	0/100.0	0/100.0						
Cicatrisation									
0—lack	0	0/0	0/0	**0.0011**	**0.0276**	0.2560	0.8312	0.5563	0.1441
1—small degree	46.7	0/0	0/0						
2—medium	53.3	100.0/100.0	25.0/100.0						
3—significant degree	0	0/0	75.0/0						

Statistically significant results are given in bold.

^a^Intensity of chronic inflammatory infiltrate measured with the number of lymphoid mononuclear cells.

There were no signs of significant passive hyperemia or purulent infiltration around the holdfast devices in Group I. The atrial surface from the clamped side was covered with a single layer of endothelial cells; furthermore, there was mature granulation tissue and chronic nonspecific inflammatory infiltrate composed of lymphoid cells without foreign body giant cells, both in-between and around the tubes of the holdfast devices. Over large surface areas, granulation tissue was partially replaced with collagenized fibrous tissue. The inner side of an appendage was filled with organized blood clots, whereas newly formed granulation tissue characterized by fibroblast proliferation was visible on the surface.

**Figure 8:**
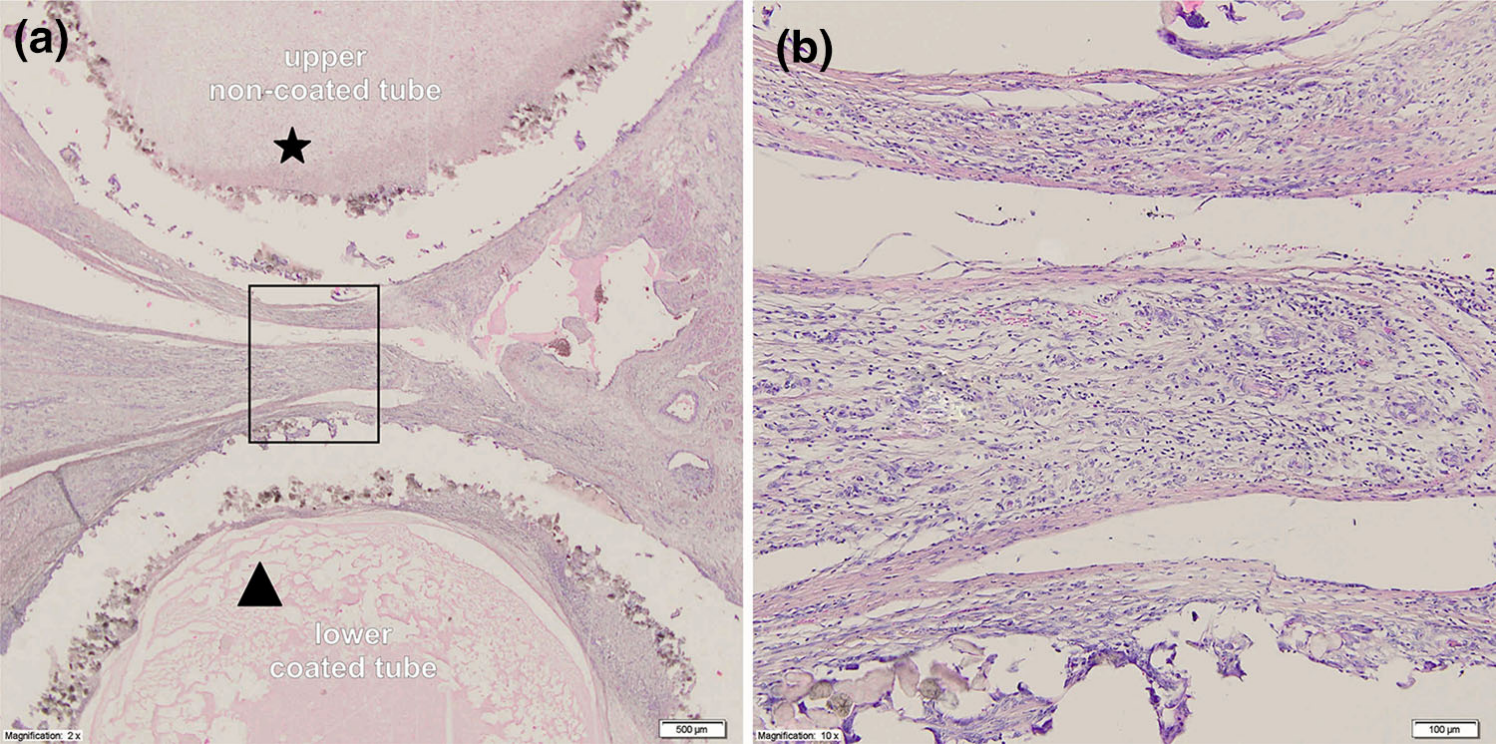
Microscopic view of the intersection through a holdfast device coated with a vascular implant used in group II. (**a**) Cross section through the device and the area between tubes of the holdfast device in group II. The the upper tube (*) was not coated with vascular implant and polyamide powder could be seen with an area of cicatrization. The lower tube (triangle) is coated with vascular implant and significant chronic inflammatory infiltrate is visible. (**b**) Higher magnification of the studied area with visible large number of giant cells.

In Group II which was subject to 14 days of incubation, there were no crucial differences observed in the level of hyperemia (lack or small, *p* > 0.05), cicatrisation (*p* > 0.05) or purulent infiltration (mainly lack, *p* > 0.05) between both tubes of the holdfast device (coated vs. not coated with arterial graft). We observed significantly more intense chronic inflammatory infiltrate and many more foreign-body giant cells around the fragment of the holdfast devices supplemented with a polyester graft (Fig. 8, *p* < 0.05). Like in Group I, no foreign-body giant cells around the fragment of the uncoated polyamide holdfast device was reported. When comparing histological features of the tissue around the coated tube in Group II with 14 days of incubation to uncoated tubes in Group I, the only observed difference was in the presence of foreign-body giant cells (*p* < 0.0001). To summarize, in Group I, connective tissue scar formation was observed without a clear inflammatory reaction, whereas in holdfast devices coated with a polyester vascular graft (Group II), there was a clear influence of this material on the heart tissue; a scar was formed around the polyester graft, and there was chronic inflammatory infiltrate with foreign-body giant cells penetrating the material.

In the animals from Group II, which had a prolonged 90 days incubation time, there was similar intense chronic inflammatory infiltration, however a greater degree of scar collagenization and cicatrisation was observed when compared to the standard 14 days Group II swines (*p* < 0.05).

## Discussion

When new biomaterials are used in any living organism, it is crucial to verify whether they can be biocompatible with the surrounding tissues to prevent any pathological postoperative reactions.^11^,^12^,^25^,^28^ Two elements should be considered: biological safety and biofunctionality. Firstly, this means the material should be tested to ensure that it does not cause any harm. Secondly, the material/device should be studied to ensure its properties function as they were designed.^3^,^11^ The host response to biomaterials has been studied for decades.^11^,^28^,^29^ Recently, there has been particular interest in the quest to finding a technical solution of obliterating the LAA, by inserting a device with biocompatible materials within the human body. This idea has been a challenge for many scientific centers.^1^,^12^,^28^,^29^ The point of contact between the material and native tissue is the most important place at which the intracellular sequence initiates the auto- and paracrine response of the host’s tissue. Therefore, the material’s surface structures, both spatial and chemical, are crucial elements for reducing the undesirable host immune response.^27^

In our previous study we have demonstrated the safety and efficacy of the 3D printed novel LAA exclusion device within an animal model, which showed rapid closure times, reduced rates of bleeding, tissue tearing and proper fibrous scar formation.^10^ Furthermore, we introduced the various possibilities and novelties associated with the manufacturing methods. Thanks to the use of 3D printing, our device could reduce the time needed for elaborate design and production. This technique could bring unique opportunities to individualize each product depending on the needs of the patient. Finally, the proposed approach would be deemed acceptable in terms of overall device cost.^10^ The estimated price of one 3D print LAA holdfast device would come to about 50 Euro. Sterilization and packaging of one device would cost about 5 Euro. This would be significantly lower than the available devices for LAA exclusion, whose costs vary from 2000 to 4000 Euro per set (e.g. AtriClip LAA Exclusion System). Moreover, the developed LAA holdfast device would have a great advantage over staplers that could be placed on the base of the LAA. Unfortunately, the latter may damage the tissue and interrupt the continuity of the left atrial wall, a phenomenon which has not been observed in printed holdfast devices. These devices would also not be as flexible as the new device we are proposing (since the staples cannot be repositioned).

This study emphasized the favorable biocompatibility of the polyamide powder (PA 2200) used in 3D printing Selective Laser Sintering (SLS) technology when compared to a well-known implantable material in humans (Vascutek Gelsoft Prosthesis, Terumo, Scotland, UK). No significant differences in terms of hyperemia and scarring were observed in between both materials, however more intense chronic inflammatory infiltrate was present around the device coated with polyester. The uncoated polyamide caused the formation of a connective tissue scar with no apparent inflammatory reaction, but an inflammatory reaction in the heart tissue was observed when it was covered with polyester. The reaction to a foreign body was connected to unspecific protein adsorption, the immune response and the presence of inflammatory cells under physiological conditions, and all of these changes were intended to protect and isolate the body from a foreign invasion.^11^,^25^,^29^ The degree of the organism’s immune reaction depends on a product’s properties, including its structure, shape, chemical composition, porosity and coarseness as well as the contact duration, material degradation and sterility.^12^,^25^ Therefore, based on our results, we can clearly state that the reported holdfast device meets all the biocompatibility criteria specified in PN-EN ISO 10993-6_2009E recommendations.

There are several limitations to this preclinical study. The study groups were relatively small, with only four subjects with a maximum of 90 days incubation. In addition, we did not analyze the effects of the holdfast device on the serum biochemistry levels. Thus, serum natriuretic peptide, troponins, creatine kinase and inflammatory response proteins were not measured. Such measurements could allow a better holistic understanding of the body’s defense responses and could assess the impact of the holdfast device on the condition of the heart. Regardless, our data did not show any macroscopic or microscopic pathological responses to the device and no clinical signs of heart failure in all animals. The surface of the atrium under a holdfast device was smooth and shiny and lacked clots, atrophy or necrosis. Our study was performed exclusively on healthy animals (with structurally unchanged heart atria), and hence evaluation using animal model with atrial fibrillation could be required to confirm our results. Finally, the appendages of older patients with various comorbidities might be more fragile and prone to tissue damage/tearing. Those features also require additional assessment in future studies.

To conclude, the current study has demonstrated that the use of 3D-printing technology could be used to design and produce an inexpensive alternative for commercially available LAA exclusion devices. The foreign body reaction of the LAA holdfast device made of polyamide powder was found to be at a statistically insignificant level and was lower when compared to the polyester graft. Our device fulfils the criteria of biocompatibility to the highest degree, which makes it a suitable material for manufacturing LAA holdfast devices. This study proves that our technology may be applied for quick, innovative and individualized production of medical implants.
